# Sterility and Sexual Competitiveness of Tapachula-7 *Anastrepha ludens* Males Irradiated at Different Doses

**DOI:** 10.1371/journal.pone.0135759

**Published:** 2015-08-14

**Authors:** Dina Orozco-Dávila, Maria de Lourdes Adriano-Anaya, Luis Quintero-Fong, Miguel Salvador-Figueroa

**Affiliations:** 1 Programa Moscafrut, Metapa de Domínguez, Chiapas, México; 2 Centro de Biociencias, Universidad Autonoma de Chiapas, Tapachula, Chiapas, México; New Mexico State University, UNITED STATES

## Abstract

A genetic sexing strain of *Anastrepha ludens* (Loew), Tapachula-7, was developed by the Mexican Program Against Fruit Flies to produce and release only males in programs where the sterile insect technique (SIT) is applied. Currently, breeding are found at a massive scale, and it is necessary to determine the optimum irradiation dose that releases sterile males with minimum damage to their sexual competitiveness. Under laboratory and field conditions, we evaluated the effects of gamma irradiation at doses of 0, 20, 40, 60 and 80 Gy on the sexual competitiveness of males, the induction of sterility in wild females and offspring survivorship. The results of the study indicate that irradiation doses have a significant effect on the sexual behavior of males. A reduction of mating capacity was inversely proportional to the irradiation dose of males. It is estimated that a dose of 60 Gy can induce more than 99% sterility in wild females. In all treatments, the degree of offspring fertility was correlated with the irradiation dose of the parents. In conclusion, the results of the study indicate that a dose of 60 Gy can be applied in sterile insect technique release programs. The application of this dose in the new genetic sexing strain of *A*. *ludens* is discussed.

## Introduction

The sterile insect technique (SIT) is an environmentally friendly method being used in various parts of the world for the control of insect pests [1,2]. It involves the breeding, sterilization and release of large numbers of insects into the field where it is hoped that the sterile males will compete successfully with wild males for mating with wild females [3,4]. The effectiveness of SIT is based on the efficiency with which sterile males transfer sterile sperm to wild females in a given population [1,2]. The success of a program depends largely on the ability of sterile males to find and successfully copulate with their wild counterparts [3,5]. Several studies have shown that irradiation causes a reduction in sexual competitiveness in males [6,7] and reduced sperm transfer [8]. This last observation implies that sterile males must not only be able to mate with wild females but must also transfer sufficient sperm to prevent the female from continuing to mate [9]. The ability to induce high levels of sterility with minimal deterioration in insect quality is a basic requirement for any pest management program [1].

In the case of fruit flies, the SIT has been applied to *Ceratitis capitata* (Wiedemann) [10], *Anastrepha ludens* [11], *Anastrepha obliqua* (Macquart) [12], *Anastrepha fraterculus* (Wiedemann) [13], *Anastrepha suspensa* (Loew) [14], *Bactrocera cucurbitae* (Coquillett) [15] and *Bactrocera tryoni* (Froggatt) [16]. However, its success varies depending on the irradiation dose that must be applied to each species. In species of the genus *Anastrepha*, the effects of pupal age and irradiation dose on the induction of sterility are not entirely consistent. In *A*. *suspensa*, it is documented that pupa irradiated to 50 Gy 48 hours before emergence have complete adult sterility [17]. Meanwhile, Calkins et al. [18] show that a dose of 30 Gy applied at 24–48 hours before emergence induces high levels of sterility. In the case of *A*. *ludens*, Rhode et al. [19] showed that irradiation of pupae to 40 Gy 96 hours before the emergence of adults induces 100% sterility in males. Velasco and Enkerlin [20] report that doses of 40 and 100 Gy induce 90 and 99% sterility, respectively, when pupae are irradiated 72 hours prior to the emergence of adults. Rull and collaborators [21] indicate that a dose starting at 40 Gy applied 48 hours before emergence induces 95% sterility. These results suggest that induction of sterility among species and even within the same species is variable and that it is necessary to assess the dose according to the type of irradiation. Gamma radiation from isotopic sources (cobalt-60 or caesium-137) is most often used in programs that release sterile insects, but high-energy electrons and X-rays are other practical option [22].

Regarding the best time to apply irradiation, studies on the species *B*. *dorsalis*, *B*. *cucurbitae* (Coquillett), *Bactrocera oleae* (Gmelin), *C*. *capitata*, *A*. *ludens*, *A*. *obliqua* (Macquart) and *A*. *suspensa* show that pupae irradiated 24–48 hours before the emergence of adults exhibit high levels of sterility [20,23,24,14].

In the case of *A*. *ludens*, the Tapachula-7 strain was developed to release only males. The separation of males is based on puparium color, which makes it possible to separate males (brown puparium) from females (black puparium) in the pupal stage [25]. This strain was initially subjected to assessments of competitiveness and sexual compatibility under field and laboratory conditions in Mexico [26] and Guatemala [27], where their viability of release in programs using the sterile insect technique was determined. The process is currently being scaled to massive levels [28], necessitating that the optimal dose of irradiation for releasing sterile males with minimum damage to their sexual competitiveness be determined.

## Materials and Methods

The tests were performed under field and laboratory conditions in the Moscafrut facility in Metapa de Dominguez, Chiapas, México. Laboratory tests were carried out under controlled conditions of 25 ± 1°C, 65 ± 5% relative humidity and 12:12 (L:D) photoperiod, with a light intensity of 350–400 lux. Field tests were conducted in an Ataulfo variety mango orchard (14°N 55” 08.9”, 92°W 16” 34.2”) under temperatures ranging from 24 to 29°C, with a relative humidity from 68 to 85% and a light intensity of 1283 to 130 lux at an altitude of 137 meters above sea level.

### Insects

Males of the mass-reared Tapachula-7 strain of *A*. *ludens* were obtained as pupae from the Moscafrut facility colony. Wild flies were obtained from infested white sapote fruit (*Casimiroa edulis*) and bitter orange (*Citrus aurantium*) collected in the Meseta Comiteca Tojolabal and Soconusco regions, Chiapas, México. Permissions for collection of biological materials were not required. In the case of *Casimiroa edulis* there were lots of fallen fruits that have no use and the species is not protected, nor endangered. In the case of *Citrus aurantim*, trees are found in private backyard gardens and we ask for permission to collect fallen infested fruits that were of no value for the owners. The insects we collected are pests of fruits and they are found in large numbers. Collections were not made in national parks or protected areas.

The site for the tests was the experimental orchard of the Moscafrut facility.

Forty-eight hours before the emergence of adults, 50 ml of brown (males) Tapachula-7 pupae were irradiated by a cobalt-60 source installed in a gamma irradiator (model GB-127Nordion International Inc., Ottawa, Ontario, Canada)) at a dose of 0, 20, 40, 60 and 80 Gy under hypoxia conditions [26]. The irradiated doses were estimated by the Fricke and Gafchromic methods [29]. The irradiated pupae and fertile wild flies were placed in separate cages made of a wooden frame (30x30x30 cm) covered with tulle mesh (2 mm). When fertile wild flies emerged, they were separated by sex and were kept in separate cages of the same type. Both the irradiated and wild fertile adults were fed water and a sugar (standard cane sugarGrupo Porres, Huixtla, Chiapas, México) and yeast hydrolysate enzymatic (MP Biomedicals, LLC, Santa Ana, California, EUA) mixture in a 3:1 ratio *ad libitum*. Males of both strains, Tapachula-7 and wild flies were used when they reached sexual maturity, 10 and 18 days of age, respectively.

### Experiment 1: Mating performance of males irradiated at different doses

Tests to compare the sexual performance of sterile males irradiated at different doses and wild sexually mature were performed in field cages. The field cages used in this study were 3 m in diameter by 2 m in height and were supported by a metal structure [30] with a citrus tree (*Citrus aurantim*) of approximately 1.8 m to the center of the cage. Two days before test, flies were marked with a small paper tag (2 mm in diameter) bearing a number (Arial type size 3) and glued on the fly thorax for individual identification [31]. This labelling method does not interfere with the sexual activity of the flies [32]. In each cage, 10 virgin males of each treatment (0, 20, 40, 60, 80 Gy and wild) and 60 wild virgin females were released. The number of matings was recorded from 16:00 to 20:00 hours, this time period includes the time of maximum sexual activity for this species [33]. Ten cages were evaluated using three different batches of insects.

### Experiment 2: Estimation of the optimal irradiation dose

Fifteen fertile wild females and 15 irradiated males (sexually mature) were placed in wooden cages (30x30x30 cm) for each irradiation dose (0, 20, 40, 60 and 80 Gy). A cage with 15 wild fertile pairs was used as a control. After three days of allowing the insects to mate, seven green fursellerone spheres were placed in each cage as an oviposition device, and they were replaced every 24 hours for 10 days. The 4.5 cm diameter spheres covered with parafilm were made with cold water (500 ml), fursellerone (15 g) and food coloring (1 ml).

Egg collection was carried out over a period of 10 days. For counting and incubation, eggs were placed in moist chambers (Petri boxes with black cloth moistened with a sponge) and incubated at 25°C for seven days. After that time, the number of neonate larvae hatched was recorded to estimate the percentage of fertility, which was used as a measure of sterility. Six replications were performed across three different lots of insects.

### Experiment 3: Estimation of F1 generation sterility at different irradiation doses

This study evaluated the sterility of the F1 generation whose parents were irradiated at low doses. In a wooden cage (30x30x30 cm) for each irradiation dose (0, 20, 40, 60 and 80 Gy), 5 fertile wild females were placed with 5 irradiated males, and as a control, one cage with 5 wild fertile pairs was kept.

A fursellerone sphere was placed in each cage for egg collection for 10 days. In the treatments where it was possible to retrieve neonate larvae, the larvae were placed on a damp cloth with a larval diet for development. At nine days, the larvae that survived were separated from the diet and placed in vermiculite to promote their pupation. Once the adults emerged, the percentage of flying adults and the transformation from egg to flying adults were estimated for each treatment. For treatments where it was possible to recover adults, when sexual maturity was reached, the adult flies were exposed to wild flies of the opposite sex at a 1:1 ratio to estimate sterility in males and females. Six replications across three different lots of insects were conducted.

### Data analysis

In all experiments, a completely random experimental design was applied. The data had an approximate normal distribution and were analyzed by one-way ANOVA using a general linear model and Fisher’s comparison of means. The significance value used in the tests was 95% (α = 0.05).

For the first experiment, six treatments and 10 replications were used. The experimental unit consisted of field cages. The copulation average per cage for each treatment with wild females was analyzed.

In the second experiment, the fertility data (%fertility = hatched eggs / oviposited eggs x 100) were used as a measure of sterility (%sterility = % fertility-100). Prior to analysis, fertility data were normalized using the arcsine transformation √X [34]. Six treatments and six replications were used. The experimental unit consisted of laboratory cages.

Finally, in the third experiment, fertility and fecundity data were obtained by applying the same method of analysis as in experiment 2. Prior to the analysis, survival (egg-adult flies) and fertility data were normalized using the arcsine transformation √X [34]. A linear Pearson (r) correlation was calculated to determine the correlation of fertility between parents and offspring. Minitab 16 statistical software was used in the analysis.

## Results

### Experiment 1: Mating performance of males irradiated at different doses

**Fig 1:**
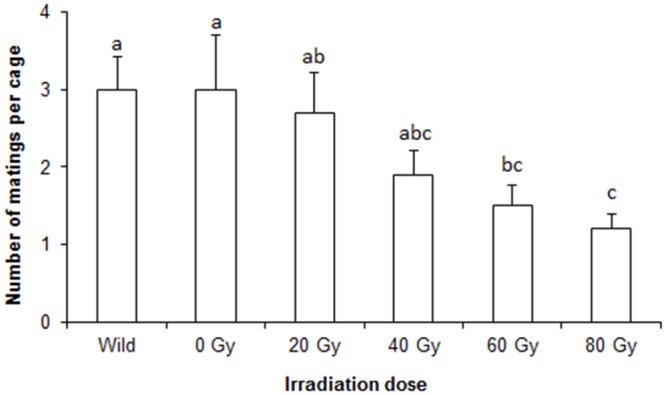
Mating behavior of wild and irradiated males with wild females. The bars symbolize the average number of mating (± standard error) recorded during evaluation of sexual performance in the field cages among irradiated males and wild females of *A*. *ludens*. Bars marked with the same letter indicate no significant differences among males (ANOVA P<0.05).

The results of the study showed significant differences in the mating behavior of wild females with males irradiated at different doses (F = 3.26; df = 5.54; P = 0.012) versus wild males (Fig 1). At 60 Gy, there were significant differences in breeding between Tapachula-7 males and wild females, while the irradiation doses of 0, 20 and 40 Gy did not result in a significant difference from wild males.

### Experiment 2: Estimation of the optimal irradiation dose

The fertility results from crosses of fertile wild females with males irradiated at different doses are shown in Table 1.

**Table 1:** Percentage of fertility and sterility induction in wild fertile females by laboratory males irradiated at different irradiation doses at a ratio of 1:1.

Gy Dose	No. eggs	No. eggs hatched	% Fertility	% Sterility
S-S	8,427	7,256	86.10±0.013a	13.90
0	10,903	8,291	76.04±0.014b	23.96
20 Gy	11,365	454	3.99±0.007c	96.01
40 Gy	12,085	108	0.89±0.007d	99.11
60 Gy	11,252	23	0.20±0.005e	99.80
80 Gy	12,880	4	0.03±0.002e	99.97

S-S = corresponds to the control cross of males and fertile wild females.

The fertility of wild fertile females decreased significantly when the irradiation dose of males increased (F = 3,622.24; df = 5.354; P<0.0001). The 60 and 80 Gy doses induced greater sterility in wild females, without a significant difference between them (P = 0.109). The 20 and 40 Gy doses induced lower sterility in wild females, with a significant difference between them (P<0.0001). There was also a significant difference between females that mated with wild males and those that mated with non-irradiated males (0 Gy dose) (P<0.0001). Doses greater than 40 Gy induce more than 99% sterility in wild females.

### Experiment 3: Estimation of sterility of the F1 generation at different irradiation doses

Survival results indicate that the egg to flying adult transformation from the F_1_ offspring of crosses of wild females with males irradiated at a low dose was significant (F = 56.43; df = 4.195; P<0.0001) (Table 2). There is a trend of lower offspring survival with higher doses of irradiation. The doses of 0 and 20 Gy resulted in increased survival of adults in the offspring of wild females with significant differences between them (P<0.001). The 40 and 60 Gy doses resulted in lower survival in the offspring of wild females without a significant difference between them. All treatments were significantly different from the offspring of males and wild females.

**Table 2:** Survival and fertility of the F1 generation from Tapachula-7 males irradiated at low doses (0, 20, 40, 60 Gy) and fertile wild females of *Anastrepha ludens* at a ratio of 1:1.

	Dose (Gy)				
	Wild	0	20	40	60
No.egg	5,892	7,239	4,942	5,520	5,195
No.larvae	3,163	1,989	320	48	9
No.pupae	3,087	1,964	306	42	8
No.adults	1,267	1,685	234	27	4
No. flying adults	934	1,552	220	23	3
Eggs to Flying Adults Transformation (%)	15.85±2.55 a	21.44±1.50 b	4.45±1.12 c	0.42±0.09 d	0.058±0.03 d
% Fertility F1 Male	71.90±2.49 a	65.88±1.12 a	52.78±4.17 b	40.28±6.57 c	1.55±1.55 d
% Fertility F1 Female	66.89±3.83ab	75.78±0.58 a	64.08± 3.49b	36.86±7.49c	12.51±3.04 d

Values with a different letter in a row indicate significant differences (p<0.05).

The difference in offspring fertility was significant for males (F = 32.08; df = 4.221; P<0.05) and females (F = 74.03; df = 4.207; P<0.05). In all treatments, a certain degree of offspring fertility was observed, and it was correlated with the irradiation dose of the males (Pearson’s = 0.71, P<0.001).

## Discussion

The results of the study indicate that irradiation dose has a significant effect on the sexual behavior of Tapachula-7 *A*. *ludens* males. In the first experiment, the results of mating behavior in field cages indicate a reduction in the mating ability inversely proportional to the irradiation dose of the males. Harmful effects of irradiation on the quality and competitiveness of flies has been documented in several species of fruit flies, including *Ceratitis capitata* [8,9], *Anastrepha obliqua* [12], *Anastrepha ludens* [21], *Anastrepha suspensa* [17] and *Bactrocera cucumis* [35]. At 60 Gy, the Tapachula-7 males show a reduction in mating behavior compared to wild males. These results are similar to those documented in a bisexual *A*. *ludens* strain, where it was shown that non-irradiated males, irradiated at 20 and 40 Gy exhibit a greater propensity for mating than males irradiated at 60 or 80 Gy [36].

These results suggest that a reduction in the irradiation dose of Tapachula-7 males could substantially improve mating performance.

In the second experiment, estimation of the optimal irradiation dose 48 hours before the emergence of adults indicates that the sterility of the flies is directly proportional to the irradiation dose evaluated. According to the results, a dose of 40 Gy in males induces 99.11% sterility in wild females. These results differ from those of studies conducted with the bisexual strain fly *A*. *ludens*, where induction of 95% sterility was documented at a dose of 40 Gy [21]. According to the development of the strain, it is apparently a genetic translocation [25] that induces a certain degree of natural sterility in the males, which increases with irradiation. The results from non-irradiated males (0 Gy) with fertile wild females indicate natural sterility of 23.96%, which represents a decrease of 10.06% compared to crosses of males and fertile wild females. The difference in sterility could be greater if it was compared with the natural sterility of a bisexual strain where fertility is approximately 90% (which is equivalent to 10% natural sterility), which has been documented [21]. These results suggest that the irradiation dose in the Tapachula-7 strain could be reduced.

Finally, in the third experiment in which we estimate the sterility of the F1 progeny of males irradiated at a low dose, we find that irradiation decreases the survival and fertility of the offspring in a manner directly proportional to the irradiation dose of the males. This result indicates a certain degree of transference of genetic damage from males to the offspring of wild females. However, this transference does not prevent a certain degree of fertility in the offspring, which could be detrimental in terms of their contribution to the next generation [37]. However, several studies document that the release of insects irradiated at low doses can be applied in certain areas where the detection of fertile insects does not activate action programs [38,12]. This observation provides the possibility of decreasing the irradiation dose, ensuring the quality of the insects, at the expense of increasing the suppression time of wild populations [21]. However, one limiting factor for using lower doses is the variation in irradiation dose depending on the precision of the irradiation source, age of the pupae, and temperature, among other factors [22]. If lower doses are used, constant evaluation of the males produced is recommended.

According to our results, it appears that a dose greater than 60 Gy induces higher sterility in wild females and that a dose less than 40 Gy causes less damage in the mating performance of males. Therefore, for practical purposes of the SIT, a dose of 60 Gy is recommended for the Tapachula-7 males of *A*. *ludens*. This result implies a moderate reduction of mating behavior in the males. However, in practice, the detrimental effects are of little importance for the efficiency of the SIT because the effects can be tolerated due to the high proportion of released insects [6], reducing the probability of offspring mating with wild populations and surviving. This reduction could result in greater induction of sterility in wild populations without presenting risks in areas where target pest populations naturally prevail and are under repression from eradication programs. Additionally, as it is a genetic sexing strain of which only the males are released, the suppression time of the pest would be shorter, resulting in more effective control and cost reductions.

In conclusion, our results suggest that a dose of 60 Gy could be applied to the new genetically sexed strain of *A*. *ludens* Tapachula-7. The high induction of sterility in wild females with apparently less damage to the sexual competitiveness of males and less probability of offspring survival benefits its use for SIT release programs.
